# Glycemic Outcomes and Feature Set Engagement Among Real-Time Continuous Glucose Monitoring Users With Type 1 or Non–Insulin-Treated Type 2 Diabetes: Retrospective Analysis of Real-World Data

**DOI:** 10.2196/43991

**Published:** 2023-01-18

**Authors:** Robert Dowd, Lauren H Jepson, Courtney R Green, Gregory J Norman, Roy Thomas, Keri Leone

**Affiliations:** 1 Department of Data Science Dexcom, Inc San Diego, CA United States; 2 Department of Medical Affairs Dexcom, Inc San Diego, CA United States; 3 Department of Global Access Dexcom, Inc San Diego, CA United States

**Keywords:** type 1 diabetes, T1D, type 2 diabetes, T2D, time in range, engagement, continuous glucose monitoring, continuous glucose monitor, CGM, diabetes management, hyperglycemia, health data

## Abstract

**Background:**

The benefits of real-time continuous glucose monitoring (RT-CGM) are well established for patients with type 1 diabetes (T1D) and patients with insulin-treated type 2 diabetes (T2D). However, the usage and effectiveness of RT-CGM in the context of non–insulin-treated T2D has not been well studied.

**Objective:**

We aimed to assess glycemic metrics and rates of RT-CGM feature utilization in users with T1D and non–insulin-treated T2D.

**Methods:**

We retrospectively analyzed data from 33,685 US-based users of an RT-CGM system (Dexcom G6; Dexcom, Inc) who self-identified as having either T1D (n=26,706) or T2D and not using insulin (n=6979). Data included glucose concentrations, alarm settings, feature usage, and event logs.

**Results:**

The T1D cohort had lower proportions of glucose values in the 70 mg/dl to 180 mg/dl range than the T2D cohort (52.1% vs 70.8%, respectively), with more values indicating hypoglycemia or hyperglycemia and higher glycemic variability. Discretionary alarms were enabled by a large majority in both cohorts. The data sharing feature was used by 38.7% (10,327/26,706) of those with T1D and 10.4% (727/6979) of those with T2D, and the mean number of followers was higher in the T1D cohort. Large proportions of patients with T1D or T2D enabled and customized their glucose alerts. Retrospective analysis features were used by the majority in both cohorts (T1D: 15,783/26,706, 59.1%; T2D: 3751/6979, 53.8%).

**Conclusions:**

Similar to patients with T1D, patients with non–insulin-treated T2D used RT-CGM system features, suggesting beneficial, routine engagement with data by patients and others involved in their care. Motivated patients with diabetes could benefit from RT-CGM coverage.

## Introduction

Real-time continuous glucose monitoring (RT-CGM) systems, which measure glucose levels in the interstitial fluid at regular intervals, benefit from ongoing improvements to sensor accuracy, improved integration with smartphones, and the inclusion of innovative functionalities such as alerts and alarms. These include high and low glucose level alerts, an urgent low soon alert, and rate of change alerts. The accuracy, functionality, and expanded insurance coverage of CGM has led to the rapidly growing adoption of this technology.

Numerous studies have demonstrated the safety and clinical efficacy of RT-CGM in individuals with type 1 diabetes (T1D) [1-4] and intensive insulin-treated type 2 diabetes (T2D) [5]. Based on this evidence, current medical practice guidelines now strongly recommend RT-CGM for all persons with diabetes treated with intensive insulin therapy, defined as 3 or more doses of insulin per day or the use of an insulin pump [6,7]. However, the guidelines also state that RT-CGM can also be considered for patients treated with basal insulin [6,7].

Studies analyzing glycemic outcomes [3-5] and effects on health care resource utilization [8] demonstrate the value of RT-CGM, but further research into the usefulness of various RT-CGM functionalities is needed. Only recently have there been studies on whether, and to what extent, patients incorporate use of the various RT-CGM functionalities and features into their daily diabetes self-management [9-11].

While current guidelines do not address RT-CGM use in patients with T2D not using insulin, some patients with non–insulin-treated (NIT) T2D do initiate use of RT-CGM with their physician’s prescription. We sought to further understand this population by analyzing their glycemic metrics, as well as their use of RT-CGM features such as alerts and retrospective data analysis. We report findings from a large, retrospective database study that quantified and compared glycemic outcomes and engagement with RT-CGM features in individuals with either T1D or NIT T2D.

## Methods

### Ethical Considerations

Historical data were from US-based Dexcom G6 users who had agreed to the privacy policy and provided consent to the use of their anonymized data for research purposes; therefore, no ethics board approval was sought.

### Study Design and Population

This retrospective, observational database analysis used anonymized data from US-based Dexcom G6 (Dexcom, Inc) users who self-identified on the G6 app as having either T1D or NIT T2D. During account initialization, those in the T1D cohort answered “Type 1” to the diabetes type question and “Yes” to the insulin use question. Those in the T2D cohort answered “Type 2” to the diabetes type question, answered “No” to the insulin use question, and did not enter any insulin doses during the observation window. Included patients had recorded use between September 1, 2021, and January 31, 2022.

### Study Device

The G6 system measures interstitial glucose concentrations and provides real-time numerical and graphical information about the current level and its rate of change. Glucose data can be viewed on a dedicated, hand-held receiver, displayed on a compatible smart device via the G6 app, or viewed on a compatible insulin pump. When the app is installed, users can access several discretionary features, including the “High Glucose” threshold alert (programmable between 120 mg/dl and 400 mg/dl), the “Low Glucose” threshold alert (programmable between 60 mg/dl and 100 mg/dl), and the “Urgent Low Soon” (ULS) alert that is triggered when a glucose value <55 mg/dl is predicted within the next 20 minutes. The app also allows users to log insulin doses, carbohydrate intake, exercise, and other health events such as stress or symptoms of hypo- or hyperglycemia.

The built-in Share feature allows users to share their glucose data and real-time alarms or alerts with up to 10 people. When “followers” download the Dexcom Follow app, they can view users’ glucose data directly from their smart device. Users and health care providers also have access to Dexcom Clarity, a suite of analytic tools and reports with up to 90 days of glucose information. The Clarity reports are available to providers through an internet portal, whereas users can access the reports on their smart device using the Clarity mobile app. The Clarity app can be programmed to allow receipt of “push” notifications, which prompt a weekly review of retrospective data and associated reports.

### Outcomes Measures

Glycemic metrics were based on recent international consensus recommendations [12]. These included the percentage of time in the 70 mg/dl to 180 mg/dl range, the percentage of time below range (either <70 mg/dl or <55 mg/dl), the percentage of time above range (either >180 mg/dl or >250 mg/dl), and the coefficient of variation. Engagement measures included screen views within the G6 app, use of Clarity, use of the data sharing feature (Share), events logged in the G6 app, and enabling or customization of alert settings.

### Analysis

Glycemic metrics and engagement with the specified system features were calculated over the 3-month observation period. Engagement with the Share feature was calculated by detecting the presence of at least 1 follower. Daily engagement with Clarity was considered if the software was used to process a patient’s data on any given day.

Outcome metrics calculated in this study considered data from the full 6-month retrospective window and aggregated over all patients within each diabetes-type segment. Glycemic metrics were obtained from the CGM-derived glucose values that update during patient use of CGM. Glycemic metrics were calculated as the mean daily percent of time spent in, above, and below range. Alert-use outcomes were reported as the percent of patients in each segment that had the given alert enabled at any point during the study window. Similarly, we reported the percentage of patients who chose to customize alert settings during the study window by either disabling G6 mobile alerts that are enabled by default (“Urgent Low Soon,” “Low,” “High,” “No Readings,” and “Out of Range”), enabling alerts that are disabled by default (“Rise” and “Fall”), as well as the percent of those who changed the default glucose threshold setting for the “High” and “Low” glucose alerts (250 mg/dl and 70 mg/dl, respectively). Screen views were calculated as the mean daily number of (G6 app) screen views in each glucose sync day for which there were any screen views. Numerical comparisons are presented here with no hypothesis testing. Statistical significance tests were not performed given that the large sample sizes of the T1D and NIT T2D groups would result in very small between-group differences considered statistically significant at conventional Type 1 error rate alpha levels.

## Results

### Glycemic Metrics

A total of 33,685 US-based users of an RT-CGM system were included in the analysis and self-identified as having either T1D (n=26,706) or T2D and not using insulin (n=6979). Overall, users with NIT T2D had a higher time in range, lower time above range, and lower time below range than users with T1D (Table 1). Their coefficient of variation, a measure of glucose variability, was also lower (Table 1).

**Table 1:** Mean glycemic metrics for users with type 1 diabetes or non–insulin-treated type 2 diabetes.

Diabetes type	TIR^a^ (%)	TAR^b^ (%)			TBR^c^ (%)			CV^d^
	70-180 mg/dl	>180 mg/dl	>250 mg/dl	<70 mg/dl		<55 mg/dl		
Type 1 diabetes	52.1	45.5	21.2	2.4		0.7	0.35	
Non–insulin-treated type 2 diabetes	70.8	28.5	7.6	0.8		0.4	0.23	

^a^TIR: time in range.

^b^TAR: time above range.

^c^TBR: time below range.

^d^CV: coefficient of variation.

### Engagement Metrics

The vast majority of users in both cohorts enabled the ULS alert, high and low glucose alerts, and the Always Sound feature (Table 2). More users customized the high glucose alert threshold than the low glucose alert threshold, and more users with T1D customized these settings than users with NIT T2D (Table 2).

Some patients chose to use the event logging features of the G6 app. Among the users with T1D, 27.2% (7272/26,706)—compared to 15.2% (1058/6979) of users with NIT T2D—logged an event of any kind (Table 3). The retrospective analysis feature (Clarity) was used by a majority in both groups (T1D: 15,783/26,706, 59.1%; NIT T2D: 3751/6979, 53.8%) (Table 3) and typically accessed within 10 days of their first data sync. In addition, use of the Share feature, which allows a trusted contact to view a user’s glycemic status on their mobile device, was higher among users with T1D (10,327/26,706, 38.7%) but still occurred in users with NIT T2D (727/6979, 10.4%) (Table 3). Finally, screen views of the G6 app were similar between the two groups (T1D: 6.6 views/day; NIT T2D: 5.8 views/day).

**Table 2:** Level of real-time continuous glucose monitoring feature use and customization.

Diabetes type	Enabled the alert or feature, n (%)				Customized alert threshold, n (%)	
	Urgent Low Soon	Low threshold	High threshold	Always Sound	Low threshold	High threshold
Type 1 diabetes (n=26,706)	25,765 (96.5)	26,326 (98.6)	25,622 (95.9)	24,222 (90.7)	16,109 (60.3)	19,669 (73.7)
Non–insulin-treated type 2 diabetes (n=6979)	6228 (89.2)	6879 (98.6)	6804 (97.5)	5967 (85.5)	3091 (44.3)	4318 (61.9)

**Table 3:** Use rates of specific real-time continuous glucose monitoring features.

Diabetes type	Event logging					Engagement or sharing		
	Any event, n (%)	Carbs, n (%)	Insulin, n (%)	Health, n (%)	Exercise, n (%)	Clarity, n (%)	Share use, n (%)	Followers^a^, mean
Type 1 diabetes (n=26,706)	7272 (27.2)	4599 (17.2)	6383 (23.9)	1616 (6.1)	1797 (6.7)	15,783 (59.1)	10,327 (38.7)	1.95
Non–insulin-treated type 2 diabetes (n=6979)	1058 (15.2)	756 (10.8)	0 (0)	314 (4.5)	437 (6.3)	3751 (53.8)	727 (10.4)	1.25

^a^The mean number of followers for those using the feature.

## Discussion

### Principal Results

In this analysis of 33,685 Dexcom G6 app users with T1D or NIT T2D, we sought to understand the level of feature engagement in these groups. We found a high degree of engagement in both cohorts in terms of enabling alerts such as the ULS alert and low and high glucose threshold alerts, as well as high levels of screen views. The higher use of data sharing in users with T1D was expected given their intensive insulin use and higher potential to contain pediatric patients. While a higher percentage of users with T1D logged events, these primarily consisted of insulin logs that users with NIT T2D did not log by definition. The number of screen views per day and Clarity usage were similar between the two groups, which suggests both groups regularly engaged with their glucose data for intraday monitoring and therapy decision-making.

### Comparison With Prior Work

CGM is associated with improved glycemic outcomes in people with T1D [1-4] and intensive insulin-treated T2D [5]. Currently, consensus recommendations include a time in range of >70% and a time below 70 mg/dl of <4% for most patients [6,12]. Higher time in range was associated with clinically significant improvements in the risk of microvascular complications [13-15] and adverse cardiovascular events [16,17]. There is a growing body of evidence demonstrating that use of CGM in individuals with T2D treated with basal insulin only or NIT is associated with improved glycemic benefits and outcomes similar to those treated with intensive insulin regimens [18-21]. Previous studies have also demonstrated an association between improved glycemic outcomes and a high level of feature usage [9,22] or persistent CGM use [9,11].

Despite the differences in diabetes therapies between individuals with T1D and NIT T2D, their similar rate of RT-CGM feature utilization is notable. However, people with T2D treated with basal insulin only or NIT are often not considered for RT-CGM, and most insurance plans do not cover RT-CGM for this population [23,24]. However, the magnitude of the glycemic benefits can be particularly high, especially for those with poorly controlled T2D. In a study of 38 patients with poorly controlled T2D (glycated hemoglobin [HbA1c]: mean 10.1%, SD 1.8%), a significant HbA1c reduction of 2.8 percentage points was observed after 3 months in the group using routine RT-CGM [21]. Similarly, a subanalysis of the MOBILE study found that participants with the highest HbA1c derived the greatest benefit from CGM (up to a 32 percentage point increase in time in range) [25] and CGM initiation in patients with poorly controlled T2D may help prevent glycemic deterioration [26]. In addition to improved glycemic control, RT-CGM is associated with reduced rates of emergency department visits and hospitalizations in patients who use insulin [27] as well as reduced diabetes-related distress and hypoglycemic concerns [28]. Additionally, large retrospective database studies of intermittently scanned CGM use in individuals treated with less intensive therapies have shown similar HbA1c improvements [20] as well as improvements in quality of life [29] and reductions in acute diabetes-related events and all-cause hospitalizations [19]. Even intermittent use of RT-CGM in individuals with T2D treated with fewer therapies has shown significant improvements in HbA1c [30,31], reductions in diabetes-related distress [32], and increased understanding of diabetes self-management concepts [31]. Moreover, evidence suggests that RT-CGM system use contributes to patients’ disease-specific knowledge [33] and may be an effective motivational tool that encourages the adoption of healthier behaviors [34-38]. This suggests that users with NIT T2D may be using the RT-CGM system and its features to monitor their glucose level in response to meals or exercise and reduce their highs and lows.

### Limitations

Limitations of this study include the use of data from only one CGM system and the all-US population, which could reduce the generalizability of our results to other systems or other countries. We also do not know why these users began using RT-CGM or their motivation level, especially those with NIT T2D who are not typically eligible for insurance coverage. In order to have a broad cohort of users, there were no restrictions with regard to CGM use rate in either cohort and, as a result, the extent of feature use could vary between avid and more sporadic users. We also do not know many patient characteristics such as the use of antidiabetic medications or whether users with T1D are using continuous subcutaneous insulin infusion (ie, an insulin pump). Metrics such as screen views could be underestimated in insulin pump users who are able to view their glucose data on the insulin pump’s interface. Additionally, the glycemic outcomes reported cannot be interpreted as causal effects of users’ engagement with the system features. Finally, the clinical relevance of the between-group differences we observed remains unknown, and we do not know the long-term effects on diabetes self-management associated with feature engagement.

### Conclusions

The high level of engagement as measured by screen views and use of features such as alerts, retrospective analysis (Clarity), and data sharing support the argument for increased CGM availability to people with NIT T2D. The RT-CGM users in our analysis were highly engaged with the various features studied.

Regardless of diabetes type and therapy regimen, users of the Dexcom G6 RT-CGM system had high levels of engagement with the system’s features. Feature use among people with NIT T2D was high and often similar to engagement levels seen in people with T1D. Improved access to RT-CGM technology should be considered as a viable option for people with diabetes who are willing to incorporate it into their treatment regimens.

## Abbreviations

CGM: continuous glucose monitoring
HbA1c: glycated hemoglobin
NIT: non–insulin-treated
RT-CGM: real-time continuous glucose monitoring
T1D: type 1 diabetes
T2D: type 2 diabetes
ULS: Urgent Low Soon
